# FK506-Binding Protein 12.6/1b, a Negative Regulator of [Ca^2+^], Rescues Memory and Restores Genomic Regulation in the Hippocampus of Aging Rats

**DOI:** 10.1523/JNEUROSCI.2234-17.2017

**Published:** 2018-01-24

**Authors:** John C. Gant, Eric M. Blalock, Kuey-Chu Chen, Inga Kadish, Olivier Thibault, Nada M. Porter, Philip W. Landfield

**Affiliations:** ^1^Department of Pharmacology and Nutritional Sciences, University of Kentucky, Lexington, Kentucky 40536, and; ^2^Department of Cell, Developmental and Integrative Biology, University of Alabama at Birmingham, Birmingham, Alabama 35294

**Keywords:** aging, calcium, cytoskeleton, FKBP12.6, microarray, ryanodine receptor

## Abstract

Hippocampal overexpression of FK506-binding protein 12.6/1b (*FKBP1b*), a negative regulator of ryanodine receptor Ca^2+^ release, reverses aging-induced memory impairment and neuronal Ca^2+^ dysregulation. Here, we tested the hypothesis that *FKBP1b* also can protect downstream transcriptional networks from aging-induced dysregulation. We gave hippocampal microinjections of *FKBP1b*-expressing viral vector to male rats at either 13 months of age (long-term, LT) or 19 months of age (short-term, ST) and tested memory performance in the Morris water maze at 21 months of age. Aged rats treated ST or LT with *FKBP1b* substantially outperformed age-matched vector controls and performed similarly to each other and young controls (YCs). Transcriptional profiling in the same animals identified 2342 genes with hippocampal expression that was upregulated/downregulated in aged controls (ACs) compared with YCs (the aging effect). Of these aging-dependent genes, 876 (37%) also showed altered expression in aged *FKBP1b*-treated rats compared with ACs, with *FKBP1b* restoring expression of essentially all such genes (872/876, 99.5%) in the direction opposite the aging effect and closer to levels in YCs. This inverse relationship between the aging and *FKBP1b* effects suggests that the aging effects arise from *FKBP1b* deficiency. Functional category analysis revealed that genes downregulated with aging and restored by *FKBP1b* were associated predominantly with diverse brain structure categories, including cytoskeleton, membrane channels, and extracellular region. Conversely, genes upregulated with aging but not restored by *FKBP1b* associated primarily with glial–neuroinflammatory, ribosomal, and lysosomal categories. Immunohistochemistry confirmed aging-induced rarefaction and *FKBP1b*-mediated restoration of neuronal microtubular structure. Therefore, a previously unrecognized genomic network modulating diverse brain structural processes is dysregulated by aging and restored by *FKBP1b* overexpression.

**SIGNIFICANCE STATEMENT** Previously, we found that hippocampal overexpression of FK506-binding protein 12.6/1b (*FKBP1b*), a negative regulator of intracellular Ca^2+^ responses, reverses both aging-related Ca^2+^ dysregulation and cognitive impairment. Here, we tested whether hippocampal *FKBP1b* overexpression also counteracts aging changes in gene transcriptional networks. In addition to reducing memory deficits in aged rats, *FKBP1b* selectively counteracted aging-induced expression changes in 37% of aging-dependent genes, with cytoskeletal and extracellular structure categories highly associated with the *FKBP1b*-rescued genes. Our results indicate that, in parallel with cognitive processes, a novel transcriptional network coordinating brain structural organization is dysregulated with aging and restored by *FKBP1b*.

## Introduction

Dysregulation of neuronal Ca^2+^ concentrations and of Ca^2+^-dependent physiological responses is among the most consistent neurobiological manifestations of mammalian brain aging (Landfield and Pitler, 1984; Michaelis et al., 1984; Gibson and Peterson, 1987; Landfield, 1987; Khachaturian, 1989; Reynolds and Carlen, 1989; Thompson et al., 1996; Verkhratsky and Toescu, 1998; Hemond and Jaffe, 2005; Murchison and Griffith, 2007; Thibault et al., 2007; Oh et al., 2010). Further, Ca^2+^ dysregulation has been associated with aging-related cognitive dysfunction in multiple species (Disterhoft et al., 1996; Thibault and Landfield, 1996; Tombaugh et al., 2005; Murphy et al., 2006; Luebke and Amatrudo, 2012; Gant et al., 2015) and evidence of Ca^2+^ dysregulation also is present in postmortem Alzheimer's disease (AD) brains and in mouse models of AD (Nixon et al., 1994; Gibson et al., 1996; Stutzmann et al., 2006; Kuchibhotla et al., 2008; Overk and Masliah, 2017).

Little is known about the mechanisms underlying aging-related Ca^2+^ dysregulation, although a strong candidate mechanism, disruption of FK506-binding protein 12.6/1b (*FKBP1b*), has emerged recently. *FKBP1b* is a member of the FKBP family of immunophilins (Kang et al., 2008) and in muscle cells is an established negative regulator of intracellular Ca^2+^ release from ryanodine receptors (RyRs) (Zalk et al., 2007; Lehnart et al., 2008; MacMillan and McCarron, 2009).

Recently, we found that *FKBP1b* negatively regulates Ca^2+^ release from RyRs in brain neurons as well and, additionally, inhibits Ca^2+^ influx via membrane L-type Ca^2+^ channels (Gant et al., 2011, 2014, 2015). Selective knock-down of *FKBP1b* in the hippocampus of young rats recapitulates the Ca^2+^ dysregulation aging phenotype of enlarged RyR-dependent Ca^2+^ potentials and currents (Gant et al., 2011). Further, chronic-stress-induced *FKBP1b* disruption is associated with cognitive dysfunction (Liu et al., 2012). In addition, short term (ST) virally mediated overexpression of *FKBP1b* in rat hippocampus reverses aging-related elevation of Ca^2+^ transients and spatial memory deficits (Gant et al., 2015). Moreover, hippocampal *FKBP1b* protein and gene expression declines with normal aging in rats (Kadish et al., 2009; Gant et al., 2015) and in early-stage AD (Blalock et al., 2004). These findings suggest that *FKBP1b* is a key regulator of neuronal Ca^2+^ homeostasis and cognitive processing that is disrupted during aging.

Transcriptional and translational processes, notably for the activity-regulated, cytoskeletal-associated protein (Arc), also have been linked to memory, synaptic growth, and dendritic remodeling (Steward et al., 1998; Guzowski et al., 2000; Schafe and LeDoux, 2000; Ploski et al., 2008; Lee and Silva, 2009; Alberini and Kandel, 2014; Fletcher et al., 2014), but it is unclear whether they are modulated by the *FKBP1b* network. Electrical activity at synapses and plasmalemmal membranes can trigger genomic responses via multiple Ca^2+^ signaling cascades or Ca^2+^-dependent transcription factors (Graef et al., 1999; Gall et al., 2003; Greer and Greenberg, 2008) and elevated intracellular Ca^2+^ concentrations induce electrophysiological and structural signs of deterioration (Scharfman and Schwartzkroin, 1989; Bezprozvanny and Mattson, 2008) that activate genomic pathways involved in cell death. In addition, *FKBP1b* and its protein isoform, FKBP1a, regulate non-RyR-dependent pathways, including the mechanistic target of rapamycin (mTOR) pathway, which modulates brain-derived neurotrophic factor (BDNF) and other transcriptionally active factors (Binder and Scharfman, 2004; Hoeffer et al., 2008; Lynch et al., 2008). These multiple pathways of potential downstream regulation make it important to determine whether and how the Ca^2+^ regulator *FKBP1b* interacts with plasticity-associated transcriptional processes.

Here, we used a multidisciplinary approach to test the hypothesis that *FKBP1b* overexpression also counters selective aging-related alterations in transcription. In addition, to determine whether long-term (LT) *FKBP1b* overexpression is safe and efficacious and may be a candidate preventive therapy, we compared ST hippocampal *FKBP1b* overexpression with LT *FKBP1b* overexpression, which is initiated in midlife when memory impairment first begins to emerge (Forster and Lal, 1992; Gallagher and Rapp, 1997; Markowska, 1999; Wyss et al., 2000; Bizon et al., 2009; Scheinert et al., 2015). Together, the results indicate that LT and ST virally mediated *FKBP1b* overexpression can prevent and reverse, respectively, important aspects of aging-related brain decline.

## Materials and Methods

All experiments and procedures were performed in accordance with the University of Kentucky guidelines and were approved by the Animal Care and Use Committee. Hippocampal overexpression of *FKBP1b* was induced using methods and doses similar to those we described and validated previously (Gant et al., 2015). Briefly, bilateral injection of adeno-associated virus (AAV) vector harboring the transgene for *FKBP1b* under control of the calmodulin-dependent protein kinase II (CaMKII) promoter (AAV2/9.CaMKII 0.4.rat*FKBP1b*.RGB; or AAV-*FKBP1b*) into the CA1 region of the hippocampus. A control vector harboring the transgene for enhanced green fluorescent protein (eGFP) (AAV2/9.CaMKII 0.4.eGFP.RGB, or, AAV-eGFP) was administered with the same procedure to a vector control group of aging rats from the same cohort. The AAV vectors were constructed at the University of Pennsylvania vector core (Philadelphia, PA).

A total of 52 male F344 rats completed behavioral training and were used for this study. The rats were divided into 4 treatment groups: (1) young controls receiving no injections (YC; *n* = 10, 4–5 months of age at receipt); (2) LT aged vector control (AC; *n* = 13, bilateral injections of AAV-eGFP 1.86e13 gene copies (GC)/ml, 2 μl per side; at 13 months of age; (3) ST *FKBP1b* (ST) (*n* = 13, bilateral injections of AAV.*FKBP1b* 1.99e12 GC/ml, 2 μl per side, at 19 months of age); and (4) LT *FKBP1b* (LT) (*n* = 16, bilateral injections of AAV-*FKBP1b* 1.99e12 GC/ml, 2 μl per side; at 13 months of age). All aged animals used in this study arrived together and were housed in our animal care facility for the same duration. During this period, two AC, two ST animals, and one LT animal could not complete the study and were euthanized because of poor health.

Infusion was accomplished using a Kopf stereotaxic instrument and Stoelting QSI microinfusion pump. After anesthesia, small holes were drilled bilaterally in the subjects' skulls and the dura was pierced. AAV constructs were infused via a 10 μl Hamilton microsyringe with a 32 gauge needle into the hippocampus at a rate of 0.2 μl/min. Stereotaxic coordinates were measured from bregma: 4.5 mm caudal, 3.0 mm lateral, and 1.7–1.9 mm depth from the brain surface based on histological pilot work.

The Morris water maze (MWM) was similar to that used in prior work (Rowe et al., 2007; Latimer et al., 2014; Gant et al., 2015). Briefly, it consisted of a 190-cm-diameter black round tub filled with water (26°C). A 15-cm-diameter escape platform was placed in 1 of 4 pool quadrants 1 cm below the water. The pool was contained within a four-sided black-curtained enclosure with high-contrast lighted geometric images (90 × 90 cm) on three of the curtain walls. Contrast imaging and water maze acquisition software was used for animal positional tracking and digitizing (Columbus Instruments) and measures of latency, path length to platform location, and annulus crossings were recorded.

During the first 4 d (training), the escape platform remained in the same quadrant and position. Each training day consisted of three trials. On each trial, the subject was started in a different nongoal quadrant and the order of the starting quadrant was changed each day. Rats were given 60 s to find the platform and were allowed to stay on the platform for an additional 30 s. If the platform was not found after 60 s, then the rat was gently guided to the platform and allowed to stay there for an additional 30 s. On day 5 (reference memory probe), the platform was removed and the subjects were started in the quadrant opposite the goal quadrant and allowed to swim for 60 s.

On day 8, the platform was placed in a new quadrant location. The subjects were then given 3 trials, 1 from each nongoal quadrant, to learn the new platform location (reversal training). On day 9 (reversal memory probe), the platform was removed and subjects were allowed to swim for 60 s. On day 10, visual acuity and locomotor ability were assessed with the platform made visible by raising it 1 cm above the water surface and hanging a bright white contrasting marker 6 inches above the platform location. The subjects were again given three trials from each of the three nongoal quadrants to find the platform.

Three days after the visual acuity task, the brains were harvested for qRT-PCR, gene chip, and immunohistochemistry studies as in prior work (Kadish et al., 2009; Searcy et al., 2012; Latimer et al., 2014; Gant et al., 2015). Animals were anesthetized with pentobarbital (Fatal Plus, 50 mg/kg, i.p.). After perfusion with 150 ml of cold 0.9% saline, the brains were removed and hemisected. For qPCR and gene chip studies, the dorsal hippocampus was removed from one hemisphere. This tissue was placed in RNase-free sample tubes and stored at −80°C until further use. For immunohistochemistry studies, the other hemisphere was postfixed overnight in 4% paraformaldehyde, cryoprotected by submersion in 15% sucrose/PBS solution, and then placed in antifreeze/30% sucrose solution for storage until sectioning.

Immunohistochemistry (IHC) methods were similar to those we have described previously (Kadish and Van Groen, 2003; Gant et al., 2015). Coronal sections (30 μm) were cut on a freezing sliding microtome. The following primary antibodies were used for overnight incubation: rabbit anti-FKBP 12.6 (1:500, sc-98742; Santa Cruz Biotechnology) and mouse Microtubule Associated Protein-2 (MAP2, 1:500, MAB3418; Millipore). After incubation, sections were rinsed and transferred to the solution containing appropriate biotinylated secondary antibody for 2 h and then rinsed and transferred to the solution containing ExtrAvidin for 2 h. Sections were then incubated for 3 min with Ni-enhanced DAB solution. To obtain similarly stained material, sections from all animals were stained simultaneously in the same staining tray. The immunostained sections of the dorsal hippocampus were digitized using an Olympus DP73 camera and the resulting images were analyzed using ImageJ. For optical densitometric analysis of MAP2 immunohistochemistry, two sections of the apical dendritic layer (stratum radiatum) of hippocampal CA1 pyramidal neurons were measured per animal. Investigators were blinded to animal number and condition and all photomicrographs were taken with the same settings of the DP73 camera. The staining pattern of the anti-FKBP 12.6/1b antibody was highly similar to that seen in two prior studies of hippocampal *FKBP1b* with this antibody, one in which we selectively knocked down *FKBP1b* with short hairpin RNA targeting *FKBP1b* (Gant et al., 2011) and one in which we overexpressed *FKBP1b* and also compared endogenous *FKBP1b* in young versus ACs (Gant et al., 2015). In both prior studies, the topography of *FKBP1b* immunostaining was highly similar to that in the present study and the antibody clearly detected the experimental manipulations and the aging difference in *FKBP1b* expression in CA1. In addition, the vendor validates antibody specificity by Western blot analyses and we performed negative control studies by omitting the primary antibody and using only secondary in adjacent brain sections from the same subjects. These negative controls produced no staining in our rat brain tissues.

Dorsal hippocampal RNA was extracted according to standard protocols and evaluated using an Agilent Technologies Bioanalyzer. All samples were of sufficient quality and did not differ significantly among treatment groups (RNA integrity number: 9.4 ± 0.1 for all groups; *p* = 0.16, ANOVA across the YC, AC, ST, and LT groups). Extracted RNA was used for both PCR and microarray measures. For RT-PCR mRNA quantification, one-step real-time qRT-PCR was used. qRT-PCR amplification was performed as described previously (Gant et al., 2015) using an ABI Prism 7700 sequence detection system (Applied Biosystems) and a RNA-to-CT 1-step TaqMan kit (Life Technologies). All samples were run in duplicate in a final volume of 30 μl containing 25–50 ng of cellular RNA and a Taqman Fam-MGB probe (Rn00575368_m1; Life Technologies) with an amplicon spanning a 116 bp rat *FKBP1b* cDNA region. Cycling parameters for all assays were as follows: 30 min at 48°C, 10 min at 95°C followed by 40 cycles of 15 s at 95°C, and 1 min at 60°C. The RNA levels of Gapdh were used as normalization controls for RNA quantification.

Microarray procedures were similar to those in our prior microarray studies on hippocampal aging in rats (Blalock et al., 2003; Rowe et al., 2007; Kadish et al., 2009). Briefly, RNA extracted from dorsal hippocampus was labeled and hybridized to Affymetrix Rat Gene 1.0 ST arrays (one array per animal). Gene signal intensities for microarrays were calculated using the Robust Multiarray Average algorithm (Bolstad et al., 2003) at the transcript level and data were associated with vendor-provided annotation information.

**Figure 1.**
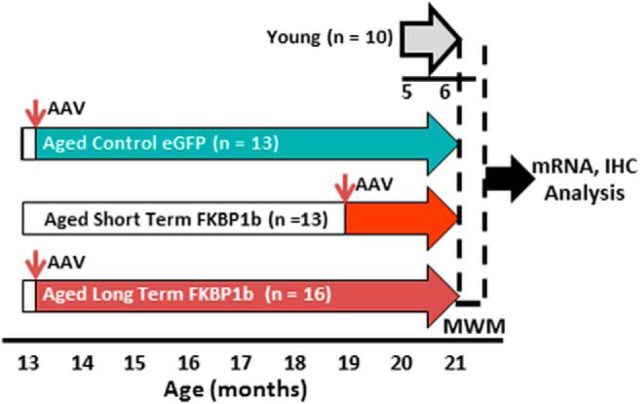
Experimental design. To compare LT with ST exposure to *FKBP1b* overexpression, aging rats were bilaterally injected in the hippocampus with AAV-*FKBP1b* at two different ages, one group at 13 months of age (LT) and one group at 19 months of age (ST) (small, vertical arrows). A third group received control vector (AAV-eGFP) at 13 months of age and a YC group received no injections. All animals were tested for spatial learning in the MWM, 3 aged groups at 21 months of age and 1 YC group at 6 months of age (total *N* = 52). Animals were killed after MWM testing. The mRNA from the dorsal hippocampus of one hemisphere was prepared for qRT-PCR and microarray analyses and the other hemisphere was postfixed for IHC.

For experimental design and statistical analyses, see Fig. 1. To test the hypothesis that expression in selective genomic systems parallels the changes in memory performance induced by aging and, in the opposite direction, by *FKBP1b* rescue, we compared YC rats and AC rats (that received bilateral injections of AAV-eGFP) with aged rats that received bilateral dorsal hippocampal injections of AAV-*FKBP1b*. The *FKBP1b*-expressing virus was microinjected into aging rats either 2 months (ST) or 8 months (LT) before testing spatial reference and reversal learning in the MWM. All three aged groups were tested together at 21 months of age along with young (5-month-old) controls (Fig. 1). The reference memory task tests the ability to learn an aspect of the task that remains constant, whereas reversal learning comprises elements of both working memory and executive cognitive function (Webster et al., 2014) and is well recognized to be particularly susceptible to impairment with aging (Bartus et al., 1979; Stephens et al., 1985). Initial statistical assessments of behavioral comparisons used ANOVA. If significance was found at the ANOVA level (α = 0.05), then protected Fisher's least significant difference (pLSD) pairwise contrasts (α = 0.05) between groups were also performed.

Microarray profiling was undertaken with the goal of identifying the *FKBP1b*-sensitive transcriptome. The *n*'s chosen for microarray studies were based on our prior work (Blalock et al., 2003; Rowe et al., 2007; Kadish et al., 2009; Blalock et al., 2010), which found that *n*'s of 5–10 per group are sufficient to identify a distinct hippocampal aging transcriptome consistently. For transcriptional profiling in the present study, a subgroup of six subjects was selected from each of the four parent treatment groups (*N* = 24 rats, one array per rat). To reduce false negatives and to maximize the transcriptional responses to elevated *FKBP1b* expression, we selected subjects for the ST and LT subgroups that showed *FKBP1b* expression levels above the medians for the parent groups. The YC and AC groups exhibited low within-group variance of *FKBP1b* expression (see Fig. 3) and these subgroups were selected at random from within their parent groups.

The Rat Gene Array used for the microarray analysis contains 29,218 total probe sets, which we filtered before statistical testing to retain only the 14,828 probe sets characterized by unique gene annotations and by signal intensity (expression) adequate to show presence of the gene (defined as unlogged signal intensity ≥40 on ≥4 arrays in the study). Expression of each of these 14,828 genes was tested by one-way ANOVA to identify genes exhibiting significant differences in expression across the four groups. Outlier values (>2 SDs of the group mean) were treated as missing values. Transcriptional profiling analyses can involve thousands of statistical comparisons and thus require assessment of multiple testing error. We estimated the error contributed by multiple testing using the false discovery rate (FDR) procedure (Benjamini and Hochberg, 1995). The FDR is the ratio of significant comparisons expected by chance to significant comparisons actually observed and is an estimate of the probability that any single significant gene in a profiling study is a false positive found due to the error of multiple testing. Therefore, confidence in profiling data increases as the FDR decreases. Confidence in microarray data is also strengthened considerably by functional category analyses, which can determine whether multiple genes in the same functional categories are coregulated (Blalock et al., 2005; Ginsberg and Mirnics, 2006).

For functional category analysis in the present study, ANOVA-significant genes were assigned to one of four expression template patterns defined by *post hoc* pairwise comparisons between groups (see Results). Overrepresented functional categories for each expression template were determined using Database for Annotation, Visualization and Integrated Discovery (DAVID) bioinformatic tools (Huang da et al., 2009) with the list of 14,828 filtered genes as a background. Raw data are available through the Gene Expression Omnibus (GSE #: 102054).

To determine the functional categories/pathways that were closely associated with each of the four template patterns, functional pathway analysis was performed as in prior work (Blalock et al., 2003, 2004; Rowe et al., 2007; Kadish et al., 2009; Chen et al., 2013). Genes assigned to each of the four template patterns were tested statistically (α = 0.05) using a modified Fisher's exact test *p-*value referred to as the “Ease Score” in the DAVID suite of bioinformatic tools (Huang da et al., 2007). This approach was used to test the Gene Ontology (GO) database (biological process, cellular component, molecular function) for categories overrepresented by genes of each template pattern. Medium classification stringency was applied. Functional annotation clustering output from DAVID was transferred to flat files and the most significant annotation from within each cluster was identified. Among these, unique functional annotations with significant Ease Scores are reported along with the number of associated pathway genes.

In a further assessment of possible contributions of chance to these large-scale analyses, the numbers of genes that were identified as belonging to a particular pattern were compared with the numbers of genes that would be expected to fall within that pattern by chance. The probability that a gene would fall into a particular pattern by chance was estimated by performing a Monte Carlo analysis in which the same statistical procedures (ANOVA test, *post hoc* pLSD, and template pattern) and statistical criteria (α = 0.05) were used, but applied to random numbers rather than actual gene signal intensity values. The Monte Carlo procedure was rerun 1000 times, with a newly generated set of random numbers in each iteration. The average number of genes found to fall within each expression pattern across 1000 iterations of random data was then used as an estimate of the number of genes expected to fall within that pattern by chance. The binomial test (α = 0.05) was used to determine whether the number actually found in the pattern exceeded significantly the number expected by chance.

## Results

### *FKBP1b* overexpression improved spatial reference and reversal memory for both LT and ST aged groups (Fig. 2)

**Figure 2.**
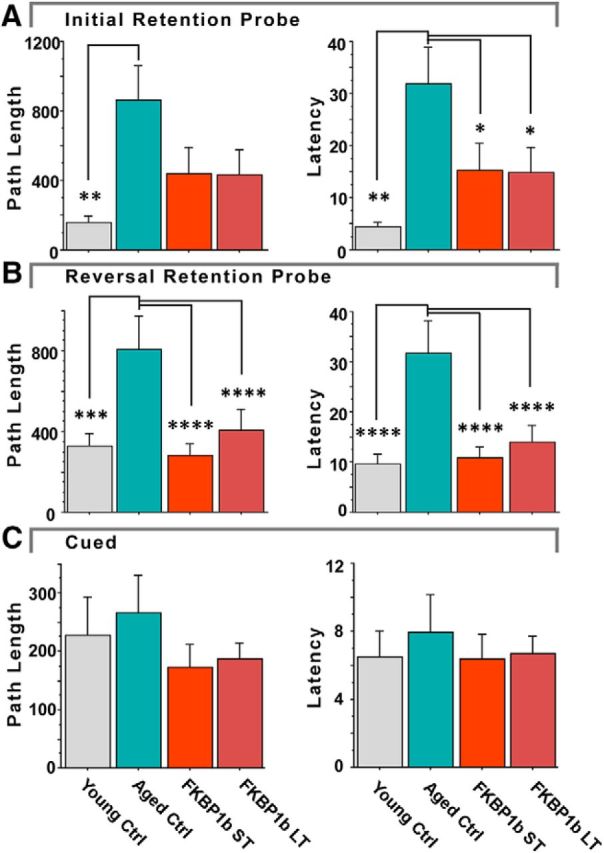
Both LT and ST *FKBP1b* overexpression countered age-related decline in spatial memory. ***A***, Reference memory probe. *FKBP1b* treatments countered age-related deficits in reference memory probe performance. ***B***, Reversal memory probe. *FKBP1b* treatments countered age-related deficits in the reversal memory probe trial. ***C***, Cued testing. With visual cues prominently highlighting location of the escape platform, no group differences were found, indicating that memory test results were not due to differences in locomotor and/or visual abilities. *p ≤ 0.05; **p ≤ 0.01; ***p ≤ 0.001; ****p ≤ 0.0001 significant pairwise contrast versus AC.

A total of 52 rats in four groups completed our spatial memory testing protocol in the MWM (10 YC, 13 AC, 13 ST, and 16 LT rats). For the 3 groups of aged AAV-treated rats, behavioral testing began at 21 months of age, either 8 (LT) or 2 (ST) months after AAV injection. Training in the MWM reference memory task was performed over 4 d, with 3 training trials per day. Over the 4 d, all groups showed acquisition of the task as indicated by a distinct decline in latency and path-length to find the platform (*F*_(11,528)_ = 11.58 *p* = 1.80e-10; *F*_(11,528)_ = 7.41, *p* = 7.20e-12, respectively, repeated-measures ANOVA). Although there was a strong trend for young animals to outperform ACs during training, no significant effects of treatment were seen in latency and path length measures over this 4 d training phase, similar to results in Gant et al. (2015).

On the fifth day of the task, the platform was removed and recall of the platform location was probed with a single retention trial (Fig. 2*A*; reference memory probe). There was a main effect of treatment group for both latency (*F*_(3,48)_ = 3.57, *p* = 0.021, ANOVA) and path length (*F*_(3,48)_ = 2.90, *p* = 0.044, ANOVA). As reported in multiple studies, the AC group exhibited significantly longer path lengths and higher latencies to find the platform compared with YC rats. In contrast, neither *FKBP1b*-treated aged group differed from YCs and both showed significantly reduced latency compared with ACs (vs ACs: LT, *p* = 0.05, ST, *p* = 0.05; YCs, *p* = 0.002, pLSD). The path length to platform results were highly similar to latency, although the differences between the *FKBP1b* groups and the ACs were only of borderline significance (vs ACs: LT, *p* = 0.078; ST, *p* = 0.08, YCs, *p* = 0.006, pLSD).

Training on the reversal memory task was conducted on the eighth day after 2 d of rest: The location of the platform was changed and the rats were given 3 trials in 1 d to learn the new location. Over the three reversal training trials, there were no significant differences in latency or path length among any of the groups, nor was there significant improvement (data not shown). On the ninth day, the platform was again removed and rats were tested for their retention of the new platform location on a single retention trial (Fig. 2*B*, reversal memory probe). There were substantial main effects of treatment on latency to platform (*F*_(3,48)_ = 9.57, *p* = 0.000046, ANOVA) and path length to platform location (*F*_(3,48)_ = 7.97, *p* = 0.0002, ANOVA). AC animals again exhibited highly significant deficits in path length and latency compared with YCs, but both ST and LT groups exhibited path length and latency scores that were highly similar to those of YCs and significantly reduced compared with AC animals (latency; AC vs: LT, *p* < 0.0001, ST, *p* < 0.0001; YC, *p* < 0.0001; path length, AC vs: LT, *p* = 0.0001, ST, *p* = 0.0001; YC, *p* = 0.0008, pLSD; Fig. 2*B*). There was also a main effect of treatment group for platform crossings during the reversal retention test (*F*_(3,48)_ = 7.37, *p* = 0.0004; ANOVA), with the YC, ST, and LT groups showing significantly greater platform crossings compared with ACs (ACs vs: YCs, *p* < 0.0001; ST; *p* < 0.025; LT, *p* < 0.01, pLSD).

On the 10^th^ day of the protocol, a cued retention test was given with visual cues highlighting the platform's location. All groups found the platform rapidly and no significant group differences were present in latency, path length, or swim speed in locating the platform (Fig. 2*C*, cued trial), indicating that aging-related changes in locomotor and visual acuity did not account for the differences in memory performance.

The ST and LT groups were statistically indistinguishable from each other on all latency and path length measures in the behavioral testing protocols. These results suggest that the reversal by ST and prevention by LT of aging-dependent memory impairment may be mediated by similar cellular mechanisms despite the differences in the duration of exposure.

### AAV-*FKBP1b* injection increased hippocampal *FKBP1b* protein and gene expression, particularly in the LT group (Fig. 3)

**Figure 3.**
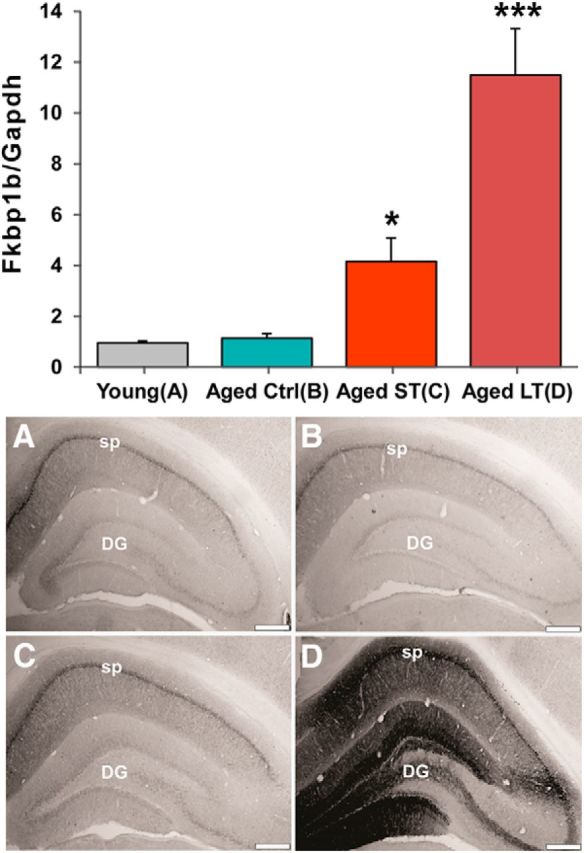
Hippocampal *FKBP1b* mRNA and protein levels were increased substantially by LT AAV-*FKBP1b* overexpression. Top, qRT-PCR quantification of hippocampal *FKBP1b* mRNA expression (*FKBP1b*/Gapdh) for each treatment group (one-way ANOVA on ranks, *p* = 0.000050; for pairwise contrast vs AC; *p ≤ 0.05; ***p ≤ 0.001). Bottom, Immunostaining for hippocampal *FKBP1b* expression. Representative photomicrographs are shown from YC (***A***), AC (***B***), aged ST *FKBP1b* (***C***), and aged LT *FKBP1b* (***D***). Note the substantial increase in *FKBP1b* expression at both the mRNA and protein levels, particularly in the LT-*FKBP1b* group. sp, Stratum pyramidale; DG, dentate gyrus. Scale bar, 500 μm.

Because the Affymetrix Rat Gene 1.0 ST microarray used in the present study does not include the probe set for *FKBP1b*, we used qRT-PCR to evaluate the effectiveness of AAV-*FKBP1b* injection for inducing *FKBP1b* expression in hippocampus. *FKBP1b*/Gapdh expression is plotted as a function of treatment group in Figure 3. There was a highly significant increase in *FKBP1b* expression (*F*_(3,47)_ = 18.449, *p* = 0.000050, ANOVA on ranks, Kruskal–Wallis). By pairwise contrast (Fisher's LSD on ranks), the increased expression was significant in ST (*p* = 0.012) and highly significant in LT (*p* < 0.001). IHC in representative animals indicated that hippocampal *FKBP1b* protein upregulation paralleled *FKBP1b* mRNA increases in AAV-*FKBP1b*-treated rats and was particularly intense in LT animals (Fig. 3, bottom).

In contrast to our prior findings (Blalock et al., 2004; Kadish et al., 2009; Gant et al., 2015), we did not observe differences in endogenous *FKBP1b* expression between the AC and YC groups (Fig. 3, top). This may be due to differences between studies in tissue dissection. In the present study, we collected tissue from whole dorsal hippocampus, whereas in prior work, we measured expression primarily in the CA1 region (Blalock et al., 2004; Kadish et al., 2009; Gant et al., 2015). Therefore, the aging effect on *FKBP1b* in CA1 might have been obscured in the present work because of dilution from less age-sensitive hippocampal regions. Further studies will be needed to fully elucidate the topographic distribution of aging changes in *FKBP1b* protein and gene expression, as well as the role of potential functional changes (Lehnart et al., 2008).

### Transcriptional profiling (Fig. 4)

**Figure 4.**
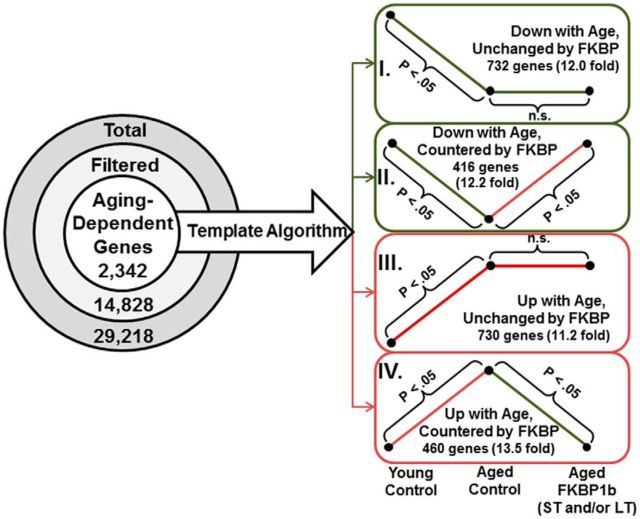
Microarray analysis flowchart. Shown are the effects of aging and *FKBP1b* on hippocampal gene transcription. Left, Total gene probe sets (29,218) were filtered to remove absent (low signal intensity) and incompletely annotated probe sets. The remaining genes (14,828) were tested by ANOVA (*p* ≤ 0.05) followed by pairwise comparison (Fisher's pLSD, ≤ 0.05 between YC and AC) to define aging-dependent genes. Right, Statistical template algorithm. Aging-dependent genes were categorized based on whether *FKBP1b* had no effect (templates I and III) or significantly countered aging's effect (templates II and IV). A total of 99.8% of aging-dependent genes were assigned to a template based on criteria described in the text. A Monte Carlo simulation (1000 iterations, see Results) was used to estimate the number of genes expected in each template by chance. The number of genes assigned to each template in the observed data was significantly greater than the number expected by chance (*p* ≤ 0.0001; binomial test; >11-fold increase for all templates). See also Figure 4-1.

10.1523/JNEUROSCI.2234-17.2017.f4-1 Figure 4-1 List of all significant genes (p ≤ 0.05, one way ANOVA) and the mean (±SEM) signal intensity values for each gene across groups, followed by ANOVA p-value. Pairwise contrast (Fisher's pLSD) results color coded for significance (0. white- n.s., 1. red- upregulated, -1. blue- downregulated). Page 1- Aging up, Unchanged by FK; page 23- Aging down, unchanged by FK; page 46- Aging up, Down by FK; page 61- Aging down, Up by FK; page 74- Unchanged by Aging. Download Figure 4-1, XLSX file

To identify genes with expression that paralleled aging and *FKBP1b*'s cognitive effects in the same animals, we first distinguished genes that changed expression with aging (i.e., differed between ACs and YCs, the aging effect). We then identified those genes among the aging-dependent genes that were also altered by *FKBP1b* overexpression (i.e., differed between LT/ST *FKBP1b* and AC, the *FKBP1b* effect). RNA from six subjects per treatment group was prepared and hybridized to Affymetrix Rat Gene 1.0 arrays. Of the ∼30,000 probe sets on the array, we filtered to retain 14,828 annotated, present genes (see Materials and Methods) for statistical analysis. One-way ANOVA (*p* ≤ 0.05) showed that 24% (3502) differed significantly across the four groups, yielding an FDR of 0.12 (see Materials and Methods). An FDR of 0.12 is quite low for a microarray study of brain aging and provides considerable confidence in these results. Reliability in microarray studies is also strengthened when functional categories are overrepresented by coregulated genes (Blalock et al., 2005; Galvin and Ginsberg, 2005; Ginsberg and Mirnics, 2006).

Among ANOVA-significant genes (3502), 2342 (67%) also differed significantly in pairwise contrast (Fisher's protected LSD; *p* ≤ 0.05) between the YC and AC groups (aging-dependent genes, “the aging effect”). Among these, the expression levels of 37% (876/2342) genes were also altered by *FKBP1b* overexpression (517 by LT, 193 by ST, 166 by both ST and LT, “the *FKBP1b* effect”) and were defined as aging and *FKBP1b*-sensitive genes (for a complete list of aging- and *FKBP1b*-sensitive genes, see Fig. 4-1). Many more of these were altered by both ST and LT (166) than would be expected by chance if ST and LT treatments acted through independent mechanisms (*p* = 4.8E-9, binomial test). These results suggest that LT and ST *FKBP1b* treatments exerted similar transcriptional effects. To determine whether this agreement between ST and LT was limited primarily to the 166 genes in the overlap or instead reflected widespread similarity among most *FKBP1b*-sensitive genes, we tested the correlation between LT and ST effects across all 876 *FKBP1b*-sensitive genes. A highly significant proportion of *FKBP1b*-sensitive genes (822/876; 93.8%) were changed in the same direction by both ST and LT (*p* ≤ 1E-12, binomial test). Further, the effect sizes of ST and LT treatment genes (expressed as log2-fold change vs AC) were strongly correlated (*r* = 0.85, *p* = 1.3E-24; Pearson's test, data not shown). These results indicate that ST and LT *FKBP1b* treatments influenced gene expression similarly. The ST and LT groups also performed nearly identically on all behavioral measures. Based on these similarities and because the genome ontology functional category analysis (see below) provides greater statistical confidence in overrepresentation of a given category with increasing numbers of genes assigned to that functional category, we combined the ST and LT lists of *FKBP1b*-sensitive genes into a single list, such that a gene was considered an *FKBP1b*-sensitive gene if it differed from ACs with ST and/or LT treatment.

Remarkably, only four of the 876 aging-dependent and *FKBP1b*-sensitive genes (*Eif3g*, *Pla2g7*, *S100β*, and *Snapc2*) exhibited exacerbation of aging effects by *FKBP1b*, whereas the other 872 changed in opposite directions with aging and *FKBP1b* treatment. Accordingly, the anomalous four genes were excluded from functional category analyses and the remaining aging-dependent genes (2338) were parsed into one of four gene expression templates (Fig. 4, right, templates I–IV), which reflected direction of a gene's expression change with aging (up or down) and whether the gene did (Fig. 4, templates II and IV), or did not (Fig. 4, templates I and III) show the counteracting effect of *FKBP1b* treatment (LT or ST vs AC; *p* ≤ 0.05, Fisher's *post hoc* LSD).

To determine whether the number of genes identified in each template was greater than expected by chance, we ran a Monte Carlo simulation using the same statistical analysis and template assignment strategy, but with randomly generated numbers substituted for signal intensity values (see Materials and Methods). The numbers of genes actually observed for each reported pattern exceeded by >11-fold the number expected by chance based on the simulation (Fig. 4; ≤ 0.00001, binomial test for each pattern), indicating a strong biological effect.

### GO functional categories associated with genes matching each of the four template patterns (Fig. 4) of aging- and *FKBP1b*-sensitive genes (Table 1)

**Table 1.** Functional categories overrepresented by genes assigned to the four expression templates reflecting aging ± *FKBP1b* sensitivity

I. Downregulated with age, unchanged by *FKBP1b*	*n*	*p*-value	II. Downregulated with age, upregulated by *FKBP1b*	*n*	*p*-value
Serine hydrolase activity	18	0.0019	Cytoskeleton	39	9.6E-04
Cellular amino acid derivative biosynthesis	9	0.0029	Passive transmembrane transporter activity	19	0.0039
Integral to plasma membrane	27	0.0050	Extracellular region	41	0.0080
Amine metabolic process	27	0.0082	Ectoderm development	8	0.0249
Tissue morphogenesis	19	0.0150	Regulation of actin cytoskeleton	6	0.0265
Positive regulation of gene expression	36	0.0341	Metalloendopeptidase activity	6	0.0335
			Regulation of MAP kinase activity	8	0.0387

III. Upregulated with age, unchanged by *FKBP1b*	*n*	*p*-value	IV. Upregulated with age, downregulated by *FKBP1b*	*n*	*p*-value
Translational elongation	36	5.9E-23	Extracellular matrix	20	1.0E-04
Lysosome	35	2.7E-13	Primary transmemb. Transporter activity	11	9.1E-04
Immune system process	76	5.0E-11	Regulation of cell motion	15	0.0010
Endosomal part	12	3.4E-06	Basolateral plasma membrane	15	0.0021
Immune effector process	21	3.7E-06	Golgi cisterna	6	0.0027
Ribosomal small subunit biogenesis	7	2.0E-05	Blood vessel development	16	0.0035
Leukocyte activity during immune response	11	2.3E-05	Membrane-bounded vesicle	29	0.0076
Antigen processing and presentation	15	4.0E-05	Response to oxygen levels	13	0.0162
Response to stress	97	7.3E-05	Response to estrogen stimulus	10	0.0246
T cell activation	15	0.0010	Membrane organization	16	0.0247
Regulation of acute inflammatory response	8	0.0020	Intracellular transport	23	0.0281
Protein kinase cascade	26	0.0036	Organelle membrane	39	0.0385
Carbohydrate binding	25	0.0045	Homeostasis of number of cells	8	0.0419
Protein amino acid phosphorylation	44	0.0056			
Response to oxidative stress	18	0.0095			
Response to lipid	7	0.0134			
Cell-cell adhesion	17	0.0324			

Shown are gene ontology category annotations overrepresented by genes exhibiting expression patterns matching one of the four templates (Fig. 4) in direction of change and sensitivity to *FKBP1b* listed in order of significance. Left: Aging effect unchanged by *FKBP1b*. Right: Aging effect countered (opposite direction change) by *FKBP1b*. (*n*, number significant genes in category; *p*-value, modified Fisher's exact test/EASE score). Also see Table 1-1.

10.1523/JNEUROSCI.2234-17.2017.t1-1 Table 1-1 Individual genes assigned to each of the functional categories that are listed in Table 1. Functional categories are grouped by template (I-IV) assignment and listed in order of significance within template. In addition to listing the pathway name (Gene Ontology Annotations), number of genes (#), and overrepresentation p-value (p-value: DAVID modified Fisher's exact test/ EASE score), the individual aging-significant genes assigned to each pathway are also listed (and hyperlinked to www.genecards.org for additional information) alphabetically to the right of each p-value. reflecting aging +/- FKBP1b-sensitivity are shown. Left: Aging effect unchanged by FKBP1b. Right: Aging effect countered (opposite direction change) by FKBP1b. (#- number significant genes in category; p-value- modified Fisher's exact test/ EASE score), followed by list of gene symbols associated with that over-represented functional category for that template. Download Table 1-1, XLSX file

Lists of genes assigned to each of the four expression templates described above (Fig. 4) were uploaded separately to DAVID (Huang da et al., 2007) and the biological process, cellular component, and molecular function gene ontologies were interrogated. In each significant cluster, the most statistically overrepresented functional category by DAVID analysis with more than three genes was retained. The retained functional categories for each of the four templates are shown in Table 1 (for individual genes associated with each identified functional pathway, see Table 1-1).

Genes downregulated with aging and unchanged by *FKBP1b* (AC downregulated vs YC, template I) overrepresented GO annotations associated with neurotransmitter metabolism and biosynthesis in neurons. Their downregulation with aging may reflect a reduction of neuronal growth and synthetic processes.

Genes upregulated with aging and unchanged by *FKBP1b* (AC upregulated vs YC, template III) very strongly overrepresented functional categories associated with upregulated translational elongation, lysosome activity, and immune signaling, which appear to reflect major turnover of proteins, cholesterol transport, and new biosynthesis of apolipoproteins by astrocytes, as well as activated neuroinflammatory signaling by microglia. Similar patterns have been seen in multiple studies (Weindruch and Prolla, 2002; Blalock et al., 2003; Rowe et al., 2007; Kadish et al., 2009; VanGuilder et al., 2011; Chen et al., 2013). Surprisingly, inflammatory responses have generally not been found to correlate closely with learning or memory (Rowe et al., 2007; Kadish et al., 2009; VanGuilder et al., 2011) and their resistance to counteraction by *FKBP1b*, seen here, extends those prior findings.

Genes downregulated with aging and upregulated by *FKBP1b* (AC downregulated vs YC, *FKBP1b* upregulated vs AC, template II) strongly overrepresented GO categories related to intracellular and extracellular structure, including: cytoskeleton, extracellular region passive transmembrane transporter activity, ectoderm development, regulation of actin cytoskeleton organization, and regulation of MAP kinase activity (Table 1), as considered further in Discussion.

Genes upregulated with aging and downregulated by *FKBP1b* (AC upregulated vs YC, *FKBP1b* downregulated vs AC, template IV) overrepresented GO category annotations related to extracellular remodeling and transporter activity, including extracellular matrix, primary transmembrane transporter activity, blood vessel development, regulation of cell motion, and membrane-bounded vesicle (Table 1), potentially reflecting extracellular reorganization, glial and endothelial cell cytokinesis, and transport of substances between activated cells.

### MAP2: downregulation with aging and restoration by *FKBP1b* (Fig. 5, Table 2)

**Figure 5.**
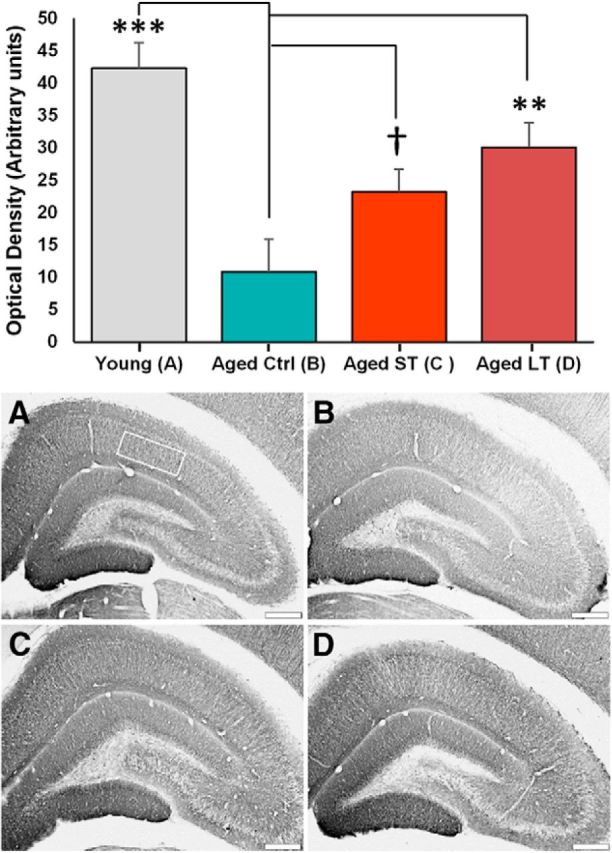
*FKBP1b* counters the age-related decrease in hippocampal MAP2 protein expression. Top, Semiquantitative measures of MAP2 IHC staining densities plotted as a function of treatment. Statistical analysis revealed a significant effect (*p* = 0.0015; one-way ANOVA) with a significant decrease from YC to AC that was countered by ST FKPB1b (**p ≤ 0.01, ***p ≤ 0.001, †n.s. trend *p* = 0.06; *post hoc* Fisher's pLSD vs AC). Bottom, Immunostaining for hippocampal MAP2 expression. Representative photomicrographs are shown from YC (rectangle defines region of quantitation; ***A***), AC (***B***), aged ST *FKBP1b* (***C***), and aged LT *FKBP1b* (***D***). Scale bar, 500 μm.

**Table 2.** *FKBP1b* counters age-related downregulation of cytoskeletal gene expression

Gene	Description	Controls		Short- term	Long-term	ANOVA *p*-value
		Young	Aged			
*Actr1a*	ARP1 actin-related prot0.1 homolog A	843 ± 10	753 ± 18	811 ± 15	801 ± 15	0.0046
*Add1*	adducin 1 (alpha)	2156 ± 15	2051 ± 38	2155 ± 20	2061 ± 24	0.0096
*Arl8b*	ADP-ribosylation factor-like 8B	3935 ± 28	3603 ± 86	3778 ± 43	3817 ± 40	0.0037
*Calm2*	calmodulin 2	8691 ± 58	8281 ± 104	8606 ± 33	8449 ± 69	0.0037
*Ccdc99*	coiled-coil domain containing 99	91 ± 2	77 ± 1	90 ± 3	81 ± 2	0.0007
*Cort*	cortistatin	296 ± 2	272 ± 8	274 ± 5	294 ± 5	0.0068
*Csrp3*	cysteine and glycine-rich protein 3	54 ± 1	43 ± 2	47 ± 2	51 ± 2	0.001
*Csta*	cystatin A (stefin A)	105 ± 4	84 ± 2	95 ± 4	104 ± 7	0.0181
*Dnai2*	dynein, axonemal, intermediate chain 2	80 ± 2	70 ± 4	73 ± 2	81 ± 3	0.0311
*Emd*	emerin	752 ± 5	707 ± 10	739 ± 9	708 ± 8	0.0015
*Gphn*	gephyrin	839 ± 10	774 ± 28	847 ± 8	810 ± 14	0.0296
*Kb23*	type II keratin Kb23	301 ± 8	175 ± 10	228 ± 28	253 ± 23	0.0027
*Kifap3*	kinesin-associated protein 3	2442 ± 26	2264 ± 23	2373 ± 24	2366 ± 20	0.0007
*Krt10*	keratin 10	73 ± 3	59 ± 2	61 ± 2	73 ± 5	0.0127
*Krt17*	keratin 17	65 ± 2	53 ± 2	62 ± 2	67 ± 2	0.0012
*Krt23*	keratin 23 (histone deacetylase inducible)	59 ± 2	52 ± 1	55 ± 2	61 ± 2	0.0117
*Krt24*	keratin 24	55 ± 1	47 ± 1	51 ± 1	51 ± 1	0.0014
*Krt28*	keratin 28	99 ± 3	87 ± 3	86 ± 2	101 ± 4	0.0037
*Krt33b*	keratin 33B	166 ± 9	122 ± 4	137 ± 8	164 ± 11	0.0064
*Krt9*	keratin 9	153 ± 3	121 ± 4	123 ± 4	149 ± 4	6.0E-06
*Krtap1–5*	keratin associated protein 1–5	66 ± 3	55 ± 3	58 ± 0	64 ± 1	0.0174
*Lmnb2*	lamin B2	121 ± 1	111 ± 3	118 ± 2	123 ± 3	0.0238
*Mk1*	Mk1 protein	61 ± 2	51 ± 2	59 ± 1	54 ± 3	0.0176
*Myoz3*	myozenin 3	359 ± 2	336 ± 8	359 ± 6	357 ± 6	0.0478
*Pfn2*	profilin 2	3277 ± 33	2970 ± 63	3134 ± 33	3138 ± 24	0.0006
*Pja2*	praja ring finger 2	4192 ± 50	3998 ± 53	4203 ± 26	4060 ± 13	0.0037
*Ppp2ca*	protein phosphatase 2 alpha	4039 ± 35	3762 ± 46	3946 ± 15	3929 ± 24	0.0001
*Ppp4c*	protein phosphatase 4, catalytic subunit	739 ± 13	648 ± 32	689 ± 12	742 ± 11	0.0063
*Prph*	peripherin	96 ± 3	82 ± 2	87 ± 2	95 ± 5	0.0309
*Sec62*	SEC62 homolog (S. cerevisiae)	1106 ± 9	1044 ± 6	1095 ± 19	1085 ± 14	0.031
*Sept4*	septin 4	2445 ± 66	2035 ± 145	2392 ± 77	2371 ± 68	0.0313
*Sept7*	septin 7	2249 ± 25	2123 ± 40	2288 ± 28	2279 ± 36	0.0115
*Shroom4*	shroom family member 4	141 ± 2	115 ± 5	126 ± 5	136 ± 6	0.0138
*Tmem200a*	transmembrane protein 200A	2344 ± 36	2001 ± 46	2072 ± 50	2303 ± 29	2.0E-05
*Tns4*	tensin 4	64 ± 1	56 ± 2	60 ± 1	66 ± 2	0.0008
*Trim32*	tripartite motif-containing 32	1640 ± 15	1482 ± 23	1494 ± 36	1608 ± 28	0.0009
*Trip10*	thyroid hormone receptor interactor 10	252 ± 6	224 ± 7	241 ± 8	261 ± 8	0.0158
*Tuba1c*	tubulin, alpha 1C	2354 ± 28	2131 ± 27	2150 ± 43	2309 ± 33	0.0003
*Ywhaz*	tyrosine 3/5-monooxygenase zeta	5666 ± 36	5491 ± 47	5655 ± 38	5506 ± 37	0.0074

Gene symbols, descriptions, mean expression level (± SEM) per group, and one-way ANOVA *p*-values are reported. Genes shown are the 39 cytoskeletal category genes identified in Table 1 (template II) that were downregulated significantly with aging and upregulated by *FKBP1b* treatment.

The “cytoskeleton” category was the most statistically significant downregulated with aging and upregulated by *FKBP1b* functional category and has been consistently identified as being age dependent in our prior studies on hippocampal gene expression associations with memory (Blalock et al., 2003; Rowe et al., 2007; Kadish et al., 2009). Accordingly, to confirm that these gene expression alterations were reflected in cytoskeletal changes at the protein level, we used semiquantitative IHC to analyze MAP2, an abundant somatodendritic MAP that reflects general microtubular structure (Fig. 5). MAP2 protein expression was significantly reduced (*F*_(3,18)_ = 7.792, *p* = 0.0015, ANOVA, **post hoc pLSD *p* ≤ 0.001) with age and rescued by LT treatment (pLSD *p* ≤ 0.01) and possibly ST treatments (borderline significant, pLSD *p* = 0.06), consistent with the view that the cytoskeletal genomic alterations are reflected in overall function and protein structure of the cytoskeleton.

### Possible downstream mediators of *FKBP1b* effects

#### Calpain-1, calcineurin, and other Ca^2+^-related genes (Table 3)

**Table 3.** *FKBP1b* counters age-related gene expression changes in key calcium-signaling pathways

Gene	Description	Controls		Short-term	Long-term	ANOVA *p*-value
		Young	Aged			
Downregulated with age, upregulated by *FKBP1b*						
*Cab39*	Calcium binding protein 39	1499 ± 15	1387 ± 30	1483 ± 16	1469 ± 19	0.0074
*Cabp4*	Calcium binding protein 4	193 ± 02	162 ± 07	170 ± 07	181 ± 04	0.0034
*Calm2*	Calmodulin 2	8691 ± 58	8281 ± 104	8606 ± 33	8449 ± 69	0.0037
*Calpns1*	Calpain, small subunit 1	2910 ± 18	2755 ± 28	2929 ± 24	2838 ± 55	0.0129
*Ppp3cb*	Protein phosphatase 3 (calcineurin), catalytic subunit, beta	3295 ± 36	2942 ± 72	3054 ± 41	3198 ± 53	0.0009
Upregulated with age, downregulated by *FKBP1b*						
*Camk1*	Calcium/calmodulin-dependent protein kinase I	231 ± 04	262 ± 11	238 ± 06	226 ± 04	0.0089
*Capn1*	Calpain 1, large subunit	191 ± 04	206 ± 04	198 ± 04	186 ± 02	0.0080
*Cib1*	Calcium and integrin binding 1 (calmyrin)	145 ± 03	173 ± 03	164 ± 03	156 ± 06	0.0012
*Orai1*	ORAI calcium release-activated calcium modulator 1	264 ± 04	279 ± 05	263 ± 03	270 ± 03	0.0389
*S100a11*	S100 calcium binding protein A11 (calgizzarin)	205 ± 11	329 ± 40	221 ± 13	228 ± 15	0.0049
*S100a6*	S100 calcium binding protein A6	95 ± 02	114 ± 05	103 ± 02	105 ± 01	0.0018

Gene symbols, descriptions, mean expression level (± SEM) per group, and one-way ANOVA *p*-values are reported. Genes presented are associated in the gene ontology category with calcium-related pathways and are altered significantly with aging and countered by *FKBP1b* treatment.

Because *FKBP1b* is a negative regulator of hippocampal neuronal Ca^2+^ transients (Gant et al., 2011, 2015), it seemed feasible that pathological Ca^2+^-related signaling during aging might be restored to young levels by *FKBP1b* overexpression. We therefore identified all Ca^2+^-related genes on the microarray and determined which showed age-related changes in gene expression that were significantly countered by *FKBP1b* treatment (i.e., met the criteria for template II or IV in Fig. 4). Although Ca^2+^-related genes as a category did not show significant overrepresentation of genes with aging changes that were countered by *FKBP1b*, the genes for two Ca^2+^-sensitive enzymes frequently associated with brain aging, neuronal plasticity, and AD, Cpn-1 (calpain-1) and Ppp3cc (calcineurin, Nixon et al., 1994; Rozkalne et al., 2011; Furman and Norris, 2014), as well as a Ca^2+^ release-activated Ca^2+^ channel (ORAI1) were altered by aging and restored by *FKBP1b* treatment (Table 3). Therefore, these Ca^2+^-related genes may be sensitive downstream mediators of some aspects of both age-dependent neuronal Ca^2+^ dyshomeostasis and restoration of regulation by overexpression of *FKBP1b*.

#### mTOR pathway genes

Genes in the mTOR pathway were also of specific interest because this pathway is inhibited by the immunosuppressant drug rapamycin via complex formation with *FKBP1b*/1a. Moreover, FKBP1a, a close isoform of *FKBP1b*, negatively regulates mTOR in the brain, even in the absence of rapamycin (Hoeffer et al., 2008). These interactions of FKBPs and mTOR appear to occur independently of *FKBP1b*/1a-dependent regulation of Ca^2+^ release from RyRs. Therefore, to determine whether mTOR pathway expression is altered by *FKBP1b* activity, we investigated mTOR pathway gene expression relative to *FKBP1b* overexpression. Twenty-four mTOR pathway genes were identified by searching the GO database and the literature (Johnson et al., 2013). Of these 24, only two, Hif1an and Nfkb1, were significantly altered with both age (upregulated) and *FKBP1b* (downregulated), showing that the mTOR pathway was not statistically overrepresented by *FKBP1b*-sensitive genes (n.s., *p* = 0.78, binomial test). Protein–protein interactions importantly regulate mTOR signaling. Nevertheless, the lack of change in mTOR pathway gene expression suggests that *FKBP1b*'s genomic effects were not mediated by the mTOR pathway.

## Discussion

The present studies provide the first evidence that *FKBP1b*, a negative regulator of intracellular [Ca^2+^], modulates a previously unrecognized genomic network regulating structural organization of the brain. In the hippocampus, this network appears to be specifically targeted and dysregulated by aging. However, as shown here, *FKBP1b* overexpression can largely reverse or prevent aging-dependent alterations in gene expression in the network. In this study, *FKBP1b* overexpression restored regulation of this network in parallel with cognitive rescue in the same aged rats. Therefore, this genomic evidence adds strong new support for the hypothesis that *FKBP1b* is a linchpin of neuronal homeostasis that functions at multiple levels, including regulation of Ca^2+^, maintenance of structural integrity, and preservation of cognitive function.

### Aging-induced changes in gene expression

As in our prior work, we found a high proportion of hippocampal genes (2342/14,928) that were altered significantly with aging (Fig. 4, the “aging effect”). Further, the expression levels of 37% of these aging-altered genes also differed significantly between AC and aged *FKBP1b* rats (876/2342 genes, the “*FKBP1b* effect”). Regardless of whether the aging effect for a given gene was upregulation or downregulation, *FKBP1b* shifted the expression levels for essentially all of these genes (872 of 876, 99.5%) back toward their YC levels. This remarkably consistent restorative action of *FKBP1b* strongly suggests that disruption of *FKBP1b* or closely associated molecules may underlie a significant portion of the aging effect for the 872 *FKBP1b*-regulated genes.

Nevertheless, deficient *FKBP1b* function does not account for all aspects of genomic change with brain aging. Of the aging-altered genes, a majority (1462/2342, ∼63%) were not affected by *FKBP1b* overexpression. Notably, those associated with upregulated glial-inflammatory processes were unaffected by FKPB1b (Table 1). Therefore, it appears that other aging processes (e.g., glial) also modulate hippocampal transcription, but operate independently from or upstream of the *FKBP1b*-sensitive genomic network.

### *FKBP1b* overexpression reveals an apparent genomic network that regulates neuronal structure and is targeted by aging

*FKBP1b*-restored genes were predominantly associated with GO functional categories related to diverse components of brain structure. In particular, “cytoskeleton,” “passive membrane transport” (ion channel proteins that add structure to lipid bilayer membranes), and “extracellular region” were the three functional categories most overrepresented by genes with expression that declined with aging and increased with *FKBP1b* (Table 1). The “cytoskeleton “category was represented by numerous genes related to actin, intermediate filaments, and microtubule assembly, whereas the extracellular region category was represented by multiple genes encoding collagens and matrix metalloproteinases, two families essential for extracellular matrix assembly/remodeling. Similarly, among genes with expression that increased with aging and declined with *FKBP1b*, the most overrepresented category was “extracellular matrix,” which, despite also focusing on extracellular space, was represented by a markedly different group of genes. The “extracellular matrix” category included genes encoding proteoglycans, growth factors, and other proteins associated with glial activation and blood vessel development (Table 1 and Table 1-1). These patterns of expression in *FKBP1b*-sensitive aging genes appear to reflect an aging-related shift in biosynthetic activity and differentiation from neuronal and extracellular structure to glial processes/compartments. The disparate structural systems altered by aging and restored by *FKBP1b* suggest the operation of a novel genomic network that coordinates structural assembly among diverse brain components. This network is particularly targeted by aging, but the disruptive effects of aging can, as shown here, be prevented or reversed by *FKBP1b* overexpression in neurons.

It seems of considerable interest that cytoskeletal remodeling in dendritic spines has been shown to play a critical role in LTP (Lynch and Baudry, 1987; Lynch et al., 2008; Briz and Baudry, 2016). In the present study, cytoskeletal gene downregulation with aging and protection by *FKBP1b* was among the major effects and was confirmed at the protein level by IHC analyses of MAP2, the primary MAP of somatodendritic compartments (Fig. 5). However, more than one *FKBP1b*-associated process likely participates in memory formation. Accordingly, defining the precise pathways linking *FKBP1b* overexpression to counteraction of age-related memory decline will require substantial further study.

The gene encoding calpain-1, a major Ca^2+^-activated, cytoskeletal-degrading protease, was upregulated with aging and downregulated by *FKBP1b* (Table 3). This is the inverse pattern of that for cytoskeletal genes and suggests that calpain-1 upregulation might mediate important aspects of downregulation and degradation of the cytoskeleton during aging. Conversely, by downregulating calpain-1 expression, *FKBP1b* overexpression may restore or maintain neuronal structural integrity.

### Efficacy and safety of ST and ST effects of *FKBP1b* overexpression

Another goal of the present study was to test whether LT *FKBP1b* overexpression in middle-aged animals could prevent the emergence of cognitive impairment as effectively as ST *FKBP1b* can reverse it (Gant et al., 2015). Results showed clearly that *FKBP1b* overexpression did not lose behavioral efficacy over extended time because both the LT and ST *FKBP1b* treatment groups substantially outperformed ACs and performed similarly to each other and to young animals (Fig. 2). These results show that cognitive impairment can be prevented by LT treatment beginning in middle-aged animals and reversed by ST treatment in aged animals.

An interesting question not addressed here is whether animals already performing at a high baseline, including young animals and the small percentage of aged animals that are unimpaired (Bäckman et al., 1996; Gallagher and Rapp, 1997; Tombaugh et al., 2002; Rowe et al., 2003, 2007; Curlik et al., 2014; Ménard et al., 2015), show improved memory with *FKBP1b*. It will be important in future studies to determine whether unimpaired animals are resistant or sensitive to the actions of *FKBP1b* on memory.

Relative to safety issues, *FKBP1b* expression was significantly higher in LT compared with ST rats (Fig. 3), indicating increased expression over the 9-month exposure or that expression is greater when initiated in middle-aged versus aged animals. Regardless, the health of LT rats appeared comparable to that of ST or age-matched vector control rats because there was only one death and no apparent morbidity among LT rats over the 9-month course of the experiment. The clinical literature also indicates that AAV-mediated gene overexpression is generally long lasting and safe in brain tissue, with an extremely low probability of inducing oncogenic or other deleterious changes in DNA (Kaplitt et al., 2007; McCown, 2010).

### Do normal-aging-induced cytoskeletal conditions model preneurofibrillary tangle pathology of AD?

Advanced age is the leading risk factor for AD (Reitz et al., 2011), suggesting that some processes that drive normal brain aging also increase susceptibility to AD. Considerable evidence indicates that Ca^2+^ dysregulation is present in both normal brain aging and AD (see Introduction) and therefore is positioned to play a role in age-dependent susceptibility to AD. Cytoskeletal dysfunction, in the form of neurofibrillary tangles is one of the hallmarks of AD pathology. It is correlated with synaptic degeneration and characterized by microtubule rarefaction and hyperphosphorylation of the MAP tau (Wiśniewski and Terry, 1973; Grundke-Iqbal et al., 1979; Masliah et al., 1991; Jicha et al., 1997; Serrano-Pozo et al., 2011; Webster et al., 2014). Here, we found that hippocampal cytoskeletal gene expression is downregulated with aging and, in parallel with cognitive function, is restored by *FKBP1b*, very possibly via Ca^2+^ signaling pathways. Accordingly, although tangles are not generally present in animal models of normal brain aging, we suggest that declining *FKBP1b* function and resulting Ca^2+^ dysregulation during normal aging in animals recapitulate brain-aging processes in humans that subtly degrade the cytoskeleton and, in susceptible individuals, eventually result in irreversible neurofibrillary tangles and AD.
